# Phylogeny and population genetic analyses reveals cryptic speciation in the *Bombus fervidus* species complex (Hymenoptera: Apidae)

**DOI:** 10.1371/journal.pone.0207080

**Published:** 2018-11-21

**Authors:** Jonathan B. Koch, Juanita Rodriguez, James P. Pitts, James P. Strange

**Affiliations:** 1 Department of Biology & Ecology Center, Utah State University, Logan, Utah, United States of America; 2 United States Department of Agriculture-Agricultural Research Services, Pollinating Insects-Biology, Management, and Systematics Research Laboratory, Logan, Utah, United States of America; 3 Australian National Insect Collection, National Research Collections Australia, CSIRO National Facilities and Collections, Canberra, Australian Capital Territory, Australia; National Cheng Kung University, TAIWAN

## Abstract

Bumble bees (*Bombus* Latrielle) are significant pollinators of flowering plants due to their large body size, abundant setae, and generalist foraging strategies. However, shared setal coloration patterns among closely and distantly related bumble bee species makes identification notoriously difficult. The advent of molecular genetic techniques has increased our understanding of bumble bee evolution and taxonomy, and enables effective conservation policy and management. Individuals belonging to the North American *Bombus fervidus* species-complex (SC) are homogenous in body structure but exhibit significant body color phenotype variation across their geographic distribution. Given the uncertainty of the genealogical boundaries within the SC, some authors have synonymized all members of the *B*. *fervidus* SC within a single taxon, while others propose an alternative two taxa hypothesis. Operating under the phylogenetic species concept, our analysis supports the hypothesis that there are two independent lineages of bumble bees within the *B*. *fervidus* SC. With the current evidence, however, it is not possible to assign valid names to either of them, because both lineages include the color phenotypes found in the original species descriptions of *B*. *fervidus* and *B*. *californicus*. Cryptic speciation does not seem to be the product of Müllerian mimicry between the clades, because diverging coloration patterns are observed when the distribution of the clades overlaps. Furthermore, within each lineage there is evidence for strong population differentiation that is correlated with geographic distribution rather than color phenotype. In our study, we demonstrate the importance of obtaining a broad sample of multiple populations when conducting lower-level phylogenetic analyses. In addition to improving our knowledge of bumble bee diversification patterns, characterizing the evolutionary history of these pollinators provides the foundation needed to guide contemporary conservation assessments and management strategies.

## Introduction

Cryptic speciation is the process in which organisms share a nearly identical phenotype but belong to different species [1]. It is a common phenomenon observed across the understudied and numerically dominant insects, and can pose a significant hurdle to effective conservation and management [2]. Biodiversity is rapidly declining on a global scale primarily due to resource extraction activities associated with economic growth and expansion. In fact, it is estimated that the contemporary extinction rate is 1,000 times higher than what has been experienced prior to the global effects of humanity’s economic and developmental activities [3]. A major impediment to the effective conservation of biodiversity includes the lack of consensus among scientists and conservation practitioners on the taxonomic resolution appropriate to a conservation or management goal. Without an operational unit that considers the ecology and evolutionary history of a species, efforts to promote species conservation will remain daunting [2].

Bumble bees (Hymenoptera: Apidae, *Bombus*) are one of the most important native pollinators of North America, contributing to the ecosystem services required by wild and economically important flowering plant species [4,5]. They are dominant pollinators of the northern hemisphere, specifically in alpine and temperate ecosystems [6,7]. Furthermore, wild bumble bee populations have been found to enhance crop productivity through effective pollination [4,8,9]. However, the global decline of wild bumble bee populations due to disease, pesticides, urbanization, and agricultural intensification have prompted state, national, and international efforts to document the diversity and distribution of these iconic bee fauna [10–12].

Concurrent efforts to conserve bumble bees are dependent on recognizing operational units, whether they are species, taxonomic, evolutionary, or otherwise [2,11,13]. These units have been useful in unveiling local biotic and abiotic factors that are specific to unique evolutionary lineages of cryptic species [2,14]. Due to the spatial cohabitation of aposematic setal coloration patterns, bumble bees have proven to be difficult to identify to species by both novice and seasoned taxonomists [15–20]. The dependence on setal coloration patterns to delineate between closely related species has caused debate on the species status of many of these taxa [15,16,20]. Contemporary phylogenetic investigations using both single and multiple genetic loci, as well as morphology-based taxonomic studies, have resolved some cryptic species complexes across bumble bee subgenera [18,19,21]. It has been demonstrated with a single gene, Cytochrome *c* oxidase I (COI), that bumble bees exhibiting nearly identical aposematic coloration patterns have been found to be separate species [22–25]. However, a lack of COI variation between species has also been detected, leading to synonymizations [6,15].

In this study, we examine the evolutionary history of the *Bombus fervidus* species-group (SC), which contains two species: *B*. *fervidus* (Fabricius, 1798) and the nominal sister taxon *B*. *californicus* Smith, 1954. These species belong to the globally distributed subgenus *Thoracobombus* [21,26]. The decline in flowering plants with long corollas due to urbanization and agricultural intensification has been implicated in the decline of European *Thoracobombus* [27,28]. Additionally, North American *B*. *fervidus* and *B*. *californicus* have been found to be declining in abundance in both wild and urban environments, relative to historic population abundance estimates [29–32]. Increased disease detection in wild populations of *B*. *fervidus* and another *Thoracobombus*, *B*. *pensylvanicus*, has been hypothesized to be a major contributor to their decline in the wild [10,33].

*Bombus fervidus* and *B*. *californicus* have been recognized to be legitimate species, based on historic and contemporary investigations using taxonomic and comprehensive phylogenetic tools [16,20,21,26]. However, the lack of strong divergence in COI, and exhibition of transitional color patterns following a continuum of variation from mostly black (*i*.*e*., *B*. *californicus*) to mostly yellow (*i*.*e*., *B*. *fervidus*) has been suggested to be evidence that they are conspecific [15,34]. *Bombus californicus* is distributed from the Pacific Coast of North America, east to the Black Hills of South Dakota [16,17,20]. Unlike *B*. *californicus*, which is distributed across a broad latitudinal gradient relative to the longitudinal range, *B*. *fervidus* has a transcontinental distribution, from the Pacific Coast to the northeastern United States [15,17,20,35]. While both species are sympatric in portions of their range in western North America, Hobbs [36] suggested that *B*. *fervidus* and *B*. *californicus* differ in nesting habitats in Canada, with *B*. *californicus* nesting in wooded areas and the foothills of southern Alberta, and *B*. *fervidus* primarily found to be in the prairies [20].

**Fig 1:**
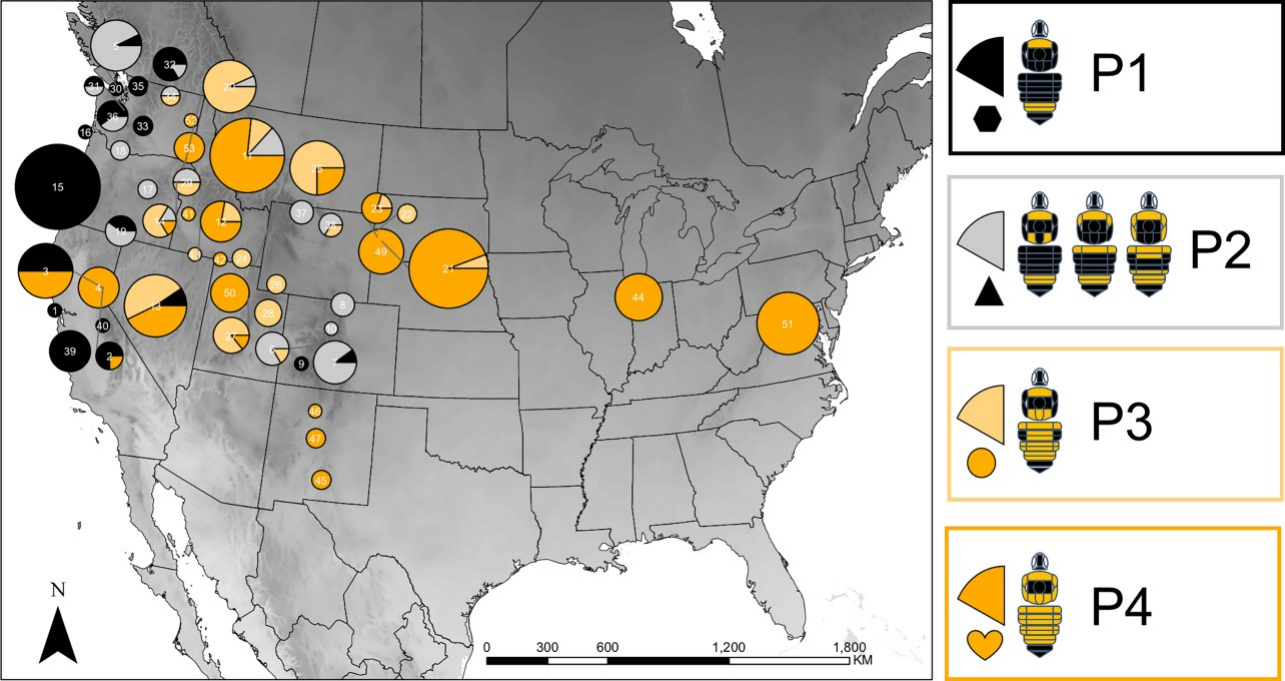
Distribution of the major phenotypes associated with *B*. *fervidus* and the nominal *B*. *californicus* in the United States. The size of each circle represents the number of specimens associated with each locality. The color of each pie slice represents the proportion of specimens exhibiting one of four phenotype (P) classes. Shapes (*i*.*e*., hexagon, triangle, circle, and heart) below each pie slice correspond to the phenotype diagrams presented in Fig 2. Phenotype diagrams are modified from Williams et al. [15]. The number at the center of each pie chart represents the field sites described in Table 1. P1-P3 = *B*. *californicus*, P4 = *B*. *fervidus*.

Setal color patterns are the principle diagnostic tool for differentiating between *B*. *fervidus* and *B*. *californicus* [16,17,20]. Historically, female *B*. *fervidus* are described to have their scutum, scutellum, metasomal tergites 1–4 with yellow setae, and metasomal tergite five with black setae (phenotype 4) (Fig 1) [17,20,37]. Conversely, female *B*. *californicus* are described to have their anterior scutum with yellow setae, scutellum with black setae, metasomal tergites 1–3 with black setae, metasomal tergite 4 with yellow setae, and metasomal tergite 5 with black setae (phenotype 1) (Fig 1) (*B*. *californicus sensu stricto*) [17,20,37]. However, since the original description of *B*. *californicus*, a number of taxa have been synonymized under *B*. *californicus*, and are now documented to be variable in black and yellow setal coloration pattern throughout their geographic distribution (phenotypes 2 and 3) (*B*. *californicus sensu lato*) [17]. In coastal populations of *B*. *californicus*, the scutellum and metasomal tergites 1–3 are with black setae (phenotype 1) [16,17,20]. However, in the intermountain west and Colorado Rockies, *B*. *californicus* populations are observed with variable banding patterns of yellow setae on their scutellum and metasomal tergites 1–3 (phenotypes 2 and 3). Historically, phenotype 3 has been recognized as a subspecies, *B*. *californicus consanguineus*, and looks very similar to *B*. *fervidus* with the exception of having two small patches of black setae on the apicolateral margins of metasomal tergite two [16,17,20,34].

Multiple taxonomic investigations of the two bumble bee species have agreed on one central idea: they are nearly impossible to separate morphologically [15,16,20,21,38]. In regards to distinguishing between *B*. *californicus consanguineus* and *B*. *fervidus*, W.P. Stephen stated in *Bumble Bees of Western North America*, “There are no morphological features in either species by which they can be distinguished, and separation is made exclusively on color pattern” [16]. In regards to distinguishing *B*. *californicus* (*sensu stricto*, phenotype 1) from *B*. *fervidus* (*sensu stricto*, phenotype 4), he went on to write, “The species is close morphologically to *B*. *fervidus* (Fabr.) and is impossible to separate structurally from that species.” [16]. Finally, W.P. Stephen citing Franklin [38], went on to state that “*californicus* and *fervidus* may eventually prove to be subspecies of a single species”. Twenty-six years later, R. Thorp led the writing of *Bumble Bees and Cuckoo Bumble Bees of California* (*Hymenoptera*: *Apidae*), and expressed a similar sentiment for the lack of variability (outside of setal color) between *B*. *californicus* and *B*. *fervidus* [20]. Furthermore, a comparison of the male genitalia between *B*. *fervidus* and *B*. *californicus* found no morphological differences [20]. However, he stated that there were distinct ecological differences between *B*. *californicus* and *B*. *fervidus* when sympatric, showing no signs of intergradation. At present, there is no biological evidence that *B*. *californicus* and *B*. *fervidus* have the capacity to breed in the wild, despite historic reports that initially proposed this hypothesis [34]. In a global systematic survey of bumble bees, Cameron et al. [26] inferred a phylogeny based on five genetic loci and found that *B*. *fervidus* and *B*. *californicus* were separated by substantial branch lengths, suggesting that they might be separate species. However, Williams et al. [15] considered that the lack morphologically diagnostic traits and COI divergence between the two species as evidence that *B*. *fervidus* and *B*. *californicus* are conspecific.

There are three major hypotheses concerning the species status of *B*. *californicus* and *B*. *fervidus*. The first hypothesis proposes that *B*. *californicus* and *B*. *fervidus* are distinct species [16,17,20,21,26]. The second hypothesis proposes that *B*. *californicus* and *B*. *fervidus* are distinct species, and produce a hybrid subspecies, *B*. *californicus consanguineus* [34]. Finally, the third hypothesis proposes that *B*. *californicus* and *B*. *fervidus* are conspecific [15,16]. Operating under the phylogenetic species concept, our goal in this study is to test all three hypotheses simultaneously. We use data from neutral and adaptive genetic loci to examine their species boundaries. We first infer a phylogeny with three mitochondrial loci: COI, 12s RNA, and 16s RNA with specimens distributed across a broad geographic range, and exhibiting diverse setal phenotypes. Next, we expand our genetic sampling effort of specimens and genotype populations using neutral microsatellite loci to examine potential hybridization and species assignment. We predict that neutral microsatellite loci will have the power to identify introgression between *B*. *fervidus* and *B*. *californicus* [39,40].

## Materials and methods

### Taxa examined

We included a total of 320 specimens associated with the *B*. *fervidus* SC, including the nominal *B*. *californicus*. We made an effort to include a diversity of setal color phenotypes associated with the *B*. *fervidus* SC (Fig 1) [15]. Exemplars of *B*. *weisi* (*Thoracobombus*) and *B*. *insularis* (*Psithyrus*) were selected as outgroup taxa based on recent *Bombus* phylogenies [21,26]. In-group taxa, exclusive to females were sampled throughout a major portion of their range in North America. We recorded setal color pattern data and locality information associated with queen and worker castes (S1 Table). We categorized specimens into four broad phenotype groups (Fig 1). These phenotype groups are based on previous taxonomic assessments of the *B*. *fervidus* SC [16,17,20]. Assignment of setal color patterns to specimens follow the schematic diagram presented in [15] and [17]. In addition to phenotypes, we assigned specimens to either *B*. *fervidus* and the nominal *B*. *californicus* following the species diagnoses from [20], [16], and [17]. In brief, *B*. *californicus* is much more variable than *B*. *fervidus*, and has been assigned three predominant setal phenotypes. Fig 1 presents the phenotypes (P) as P1, P2, and P3. *Bombus fervidus* is not considered to be variable in setal bandings, and is presented as P4 in Fig 1.

Forty-nine field sites did not require specific permissions to survey bumble bees as the surveys were conducted in public spaces that had no specific geographic protections (Table 1). Furthermore, collection permits are not necessary as members of the *B*. *fervidus* SC are not protected under any state or federal laws. Four sites did require specific permits for bumble bee surveys as they took place in U.S. National Parks. The sites and corresponding permits are as follows: Pop ID 1 = PORE-2010-SCI-0021 (Point Reyes National Seashore), Pop ID 16 = LEWI-2013-SCI-003 (Lewis & Clark National Historical Park), Pop ID 39 = PINN-2011-SCI-005 (Pinnacles National Park), and Pop ID 40 = YOSE-2004-SCI-011 (Yosemite National Park) (Table 1). Permit details may be retrieved from National Park Service Research and Reporting System (https://irma.nps.gov/rprs/). Collection data associated with specimens used for this study have been digitized and deposited in the United States National Pollinating Insect Collection at Utah State University in Logan, Utah, U.S.A (S1 Table).

**Table 1:** Survey locations of populations in the *Bombus fervidus* species complex in North America.

Pop ID	Population Code	Location Description	Latitude	Longitude	Country	State/Province	County
1	CA_Marin01	Coast Campground, Point Reyes National Seashore	38.01651	-122.85357	USA	California	Marin
2	CA_Sierra01	0.92 km SSW of Sierra Valley	39.61279	-120.42351	USA	California	Sierra
3	CA_Sierra02	1.52 km SSW Sierraville	39.57604	-120.36991	USA	California	Sierra
4	CA_Sierra03	2.33 km WNW Sierraville	39.59517	-120.39332	USA	California	Sierra
5	CAN_BC	Uplands Park, Victoria, District of Oak Bay	48.44218	-123.29772	Canada	British Columbia	
6	CO_Gunn01	2.61 km NNW Crested Butte	38.8908	-106.9951	USA	Colorado	Gunnison
7	CO_Gunn02	Swanson Lake, 2.59 km NW	38.32304	-107.4761	USA	Colorado	Gunnison
8	CO_Larimer01	Dry Gulch Rd, Estes Park	40.39179	-105.48759	USA	Colorado	Larimer
9	CO_Ouray01	Angel Creek Campground, Uncompahgre NF	38.00169	-107.69428	USA	Colorado	Ouray
10	CO_Summit01	9.54 km NW Silverthorne	39.7184	-106.1513	USA	Colorado	Summit
11	MO_Missoula01	MPG Ranch: Plot 109	46.70016	-114.03231	USA	Montana	Missoula
12	NE_Elko01	Gollaher Mtn; Chokecherry spring, 4.2km NW	41.93535	-114.50717	USA	Nevada	Elko
13	NE_Lander01	Toiyoabe Range, Birch Creek, site 5	39.38735	-117.02886	USA	Nevada	Lander
14	OR_Baker01	32.5 km NE Baker City	45.00649	-117.57936	USA	Oregon	Baker
15	OR_Benton01	Corvallis	44.5667	-123.2833	USA	Oregon	Benton
16	OR_Clatstop01	Lewis & Clark National Historic Park	46.1298	-123.8903	USA	Oregon	Clatstop
17	OR_Grant01	Billy Fields Recreation Site, 1.07 km SSW	44.3552	-119.3054	USA	Oregon	Grant
18	OR_Hood River01	0.35 km ESE of Wyeth	45.69103	-121.76563	USA	Oregon	Hood River
19	OR_Lake01	Warner Canyon Ski Area	42.23806	-120.29696	USA	Oregon	Lake
20	OR_Wallowa01	Wallowa-Whitman National Forest, 1.42km NNW of Hideaway Spring	45.70638	-117.29303	USA	Oregon	Wallowa
21	SD_Custer01	Fs Rd. 284	43.8312	-103.03775	USA	South Dakota	Custer
22	SD_Lawrence01	FS Rd.198	44.20805	-103.774533	USA	South Dakota	Lawrence
23	SD_Pennington01	Ditch Creek, West, Black Hills National Forest	44.0091	-103.831	USA	South Dakota	Pennington
24	UT_Box Elder01	Raft River Meadows	41.90004	-113.40052	USA	Utah	Box Elder
25	UT_Cache01	Logan Canyon, area 48	41.91778	-111.48035	USA	Utah	Cache
26	UT_Daggett01	3.77 km ESE Sheep Creek Lake	40.8836	-109.8066	USA	Utah	Daggett
27	UT_Wasatch01	Guardsman Pass, 7.09km SSW of Park City	40.6065	-111.555	USA	Utah	Wasatch
28	UT_Wasatch02	Timber Canyon, 3.8 km E Soldier Summit	39.9302	-111.0338	USA	Utah	Wasatch
29	WA_Asotin01	Anatone, 17 km SE	46.10825	-117.2458	USA	Washington	Asotin
30	WA_Clallam01	Dungeness Recreation Area	48.13381	-123.19755	USA	Washington	Clallam
31	WA_Clark01	Vancouver	47.47	-122.28	USA	Washington	Clark
32	WA_Island01	Kettles Trail, near Coupeville	48.34782	-121.06564	USA	Washington	Island
33	WA_Lewis01	Glenoma, 4.92 km ENE	46.53815	-122.10821	USA	Washington	Lewis
34	WA_Okanogan01	0.3 mi E Cornell Butte	48.5957	-118.8897	USA	Washington	Okanogan
35	WA_Skagit01	Concrete	48.53928	-121.74625	USA	Washington	Skagit
36	WA_Thurston01	Olympia, 2.43 km NW	47.05933	-122.92552	USA	Washington	Thurston
37	WY_Big Horn01	Medicine Mtn, 1.60 km N, Big Horn National Forest	44.80227	-107.90035	USA	Wyoming	Big Horn
39	CA_Pinnacles	High Peaks Tr; Condor Gulch Tr jct, EbyS 0.75km	36.48891	-121.18265	USA	California	San Benito
38	WY_Johnson01	Cow Camp Spring, Big Horn National Forest	44.31898	-106.94241	USA	Wyoming	Johnson
40	CA_Yos	Joes Point, 0.7 mi NNE	37.8945	-119.9493	USA	California	Tuolumne
41	ID_Ada01	Eagle, Dry Creek Cemetery, 2 km N	43.71038	-116.30246	USA	Idaho	Ada
42	ID_Cassia01	City of Rocks; Twin Sisters Peak, 3km SE	42.02338	-113.6963	USA	Idaho	Cassia
43	ID_Owyhee01	Inside Desert; Pence Butte; 10.26km SSW	42.01196	-115.33798	USA	Idaho	Owyhee
44	Indiana	PPAC3, Tarp target pest:AG	41.44395	-86.92045	USA	Indiana	Porter
45	NE_Otero01	Cloudcroft, 3.6 km NNW	32.9757	-105.7559	USA	New Mexico	Otero
46	NE_Sandoval01	Valle San Antonio	35.9749	-106.5408	USA	New Mexico	Sandoval
47	NE_Torrance01	Canon de Tajique, 4 air km NW	34.7689	-106.3285	USA	New Mexico	Torrance
49	SD_Fall River01	FS Rd. 379	43.3935	-103.751166	USA	South Dakota	Fall River
50	UT_Tooele01	Skull Valley; Salt Mtn, 10.9km NbE	40.6436	-112.68916	USA	Utah	Tooele
51	VI_Clarke01	Blandy Experiment Farm	39.065	-78.057	USA	Virginia	Clarke
52	WA_Spokane01	Spokane Airport	47.6231	-117.5133	USA	Washington	Spokane
53	WA_Whitman01	Kramer CRP	46.5829	-117.2094	USA	Washington	Whitman

Pop ID = population identification number associated with Fig 1 and Fig 3; Population Code = unique population code description; Location Description = location description of survey location, Latitude = decimal degrees latitude (WGS1984); Longitude = decimal degrees longitude (WGS1984); Country = country; State/Province = state/province; County = USA county name.

### DNA extraction, amplification, and gene sequencing

We extracted genomic DNA from the mid-leg of a specimen using a modified Chelex 10 protocol following Strange et al [41]. DNA extracted in this manner was primarily used for microsatellite genotyping (*i*.*e*., Fragment Analysis), and was not especially successful when used in PCR aimed at amplifying gene fragments >500 base pairs. In this case, we also extracted genomic DNA using the Roche High Pure Template Preparation Kit (Roche Diagnostics GmbH, Germany) to obtain high quality genomic DNA suitable for downstream amplicon sequencing.

For 64 specimens, we amplified three mitochondrial gene fragments: 489 nucleotides of 16S rRNA, 369 nucleotides of 12S rRNA, and 900 nucleotides of COI. PCR conditions and primers followed the recommendations of the published literature [21,42–44]. Briefly, PCR was carried out in a 25 μL reaction volume, containing approximately 3 μL of extracted DNA, 1x Promega (Madison, WI) reaction buffer, 0.6 mM dNTP mixture, 10 μM primer, 5 units Taq polymerase (Promega, Madison, WI) and the MgCl_2_ concentration was adjusted to 1.4 mM. 16S rRNA fragments were amplified with the primers 875-16S1F and 875-16S1R described in Cameron et al. [42] at 50°/70°C annealing and elongation temperatures, respectively. 12S rRNA fragments were amplified with the primers 12Sa-5' and 12-SLR-5' described in [21] at 48°/70°C annealing and elongation temperatures, respectively. Finally, COI was amplified with the forward primer 5'-ATAATTTTTTTTATAGTTATA-3' and the reverse primer 5'-GATATTAATCCTAAAAAATGTTGAGG-3' described in Bertsch et al. [43] from Tanaka et al. [44] at 45°/60°C annealing and elongation temperatures, respectively [43,44]. Sequencing reactions were performed for both forward and reverse DNA strands (http://etonbio.com). We edited and assembled reads, and aligned the DNA sequences with Geneious v8 (http://geneious.com [45]).

### Phylogenetic analysis

The mitochondrial genes were examined separately and combined into a single partitioned dataset (1758 nucleotides) to infer a phylogeny with a Bayesian likelihood-based approach. Models of molecular evolution for each mitochondrial locus and codon position (COI) were first investigated with PartitionFinder v1.0.1 [46]. We implemented the model HKY+Gamma for 12S and 16S, HKY+I for COI first codon position, F81 for COI second codon position and HKY for COI third codon position. The Bayesian single-gene and concatenated phylogenies were estimated with MrBayes v3.2.1 [47] using two independent runs with three heated chains and one cold chain each. The MCMC chains were run for 10 million generations with sampling every 1000 generations. Convergence diagnostics were evaluated with Tracer v1.5 [48]. Ten-percent of samples were discarded as burn-in. Trees were visualized in FigTree v1.4.0 [49].

### Microsatellite genotyping

A total of 373 bumble bees across 53 field sites were screened at 13 microsatellite loci documented in the literature: BL15, B124, BTERN01, BT28, BT10, B96, BTMS0066, B126, BTMS0062, BTERN02, BTMS0086, BTMS0044 and BTMS0059 [50–52]. PCR were performed in final volumes of 10 μL, containing approximately 1 μL of extracted DNA, 1x Promega (Madison, WI) reaction buffer, 0.6 mM dNTP mixture, 0.2–0.4 μM primer, 0.001 mg BSA, 0.4 units Taq polymerase (Promega, Madison, WI) and the MgCl_2_ concentration was adjusted to 1.4 mM. The PCR conditions for both multiplex reactions were one 3:30 min cycle at 95°C, 30 cycles of 95°C for 30 s, annealing temperature 55/58°C for 1:15 min, 72°C for 45 s and a final extension period of 15 min at 72°C. The DNA amplifications were performed with fluorescent 5’ dye-labeled primers (6-FAM, NED, VIC, or PET) and separated on an Applied Biosystems 3730xl automatic sequencer at the Center for Integrated Biology at Utah State University (Logan, UT). The allele sizes were scored manually using Geneious v8 [45]. Because we were potentially working with two different species in our study, we elected to use a universal bin set when scoring alleles for all specimens. This approach ensured that alleles were being consistently called with the appropriate microsatellites motifs with no *a priori* assumptions of species identity. Our method did not yield any ambiguous allele calls nor did we observe any “bin creep” [53], suggesting that the genotypes discovered in this study were suitable for downstream analyses.

### Population genetic analysis

A Bayesian clustering method implemented in Structure v2.3.4 [54] was used to assign individuals to populations *a priori*. This method ensured that we did not base species identifications on the setal color phenotype the specimen displayed (Fig 1). We predicted that specimens that were grouped together based on microsatellite genotypes composed distinct genetic clusters separate from specimens in other predicted groups. The Structure algorithm in this way has been found to be useful in identifying distinct genetic clusters in other studies of bumble bees with cryptic phenotypes and evolutionary histories [18].

We used the admixture model in Structure, which assumes that individuals comprise *K* unknown genetic clusters, to which an individual can be fractionally assigned. This allowed us to group specimens based on their genotype without prior delineation to a population or species. In this case, the inferred population represents a genetic cluster and would illuminate any contemporary admixture of genes. The alternative to the admixture model would be to set the modelling scheme to “no admixture” which would assume that populations are discrete, where genotypes were assigned to a genetic cluster in full (*i*.*e*., no fractional assignment). As we are testing whether *B*. *fervidus* and *B*. *californicus* are conspecific with gene flow among populations, incorporating admixture into the modelling framework would allow for fractional assignment to *K* population(s). Furthermore, the admixture model would allow us to detect if any hybridization at the microsatellite loci between the two species was evident in areas where the two color phenotypes are sympatric. We set the admixture model to run with 20,000 burn-in steps and 100,000 samples, with 10 iterations for each *K*, where *K* ranged from 1 to 10. Testing a wide range of *K* ensured that we did not bias the assignment of genotypes to only one or two species.

To determine the optimal *K* (*i*.*e*., populations/species or genetic lineages), the distributions of the probability of the data (*ln P(D)*) and *ΔK*, as described by Earl and von Holdt [55] and Evanno and vonHoldt [55], were visualized with the web-based software program Structure Harvester [55]. To account for multimodality associated with individual Structure simulations, we averaged each individual’s admixture proportions over the 10 replicates for the best *K* using Clumpp v1.1.2 [56]. Finally, in addition to Structure analyses, we combined the 13 microsatellite loci into a principal components analysis to determine if significant clustering of similar genotypes could be inferred.

After determining the appropriate species assignments and number of *K* genetic clusters, the probability of null alleles was estimated with the software program MicroChecker [57]. We then estimated pairwise linkage disequilibrium (LD) and deviations from Hardy-Weinberg equilibrium (HWE) across populations and loci with the web-based software program Genepop v 4.0.10 using default parameters [58]. Based on the genetic clusters inferred by Structure, we performed an analysis of molecular variance (AMOVA) to test for differences in genetic structure with Arlequin v3.5 [59]. We then tested for a correlation between pairwise estimates of fixation based on allele frequencies with geographic distance (Isolation by Distance) within the genetic clusters inferred from the Structure analysis with GeneAlEx v6.5 [60].

## Results

### Phylogenetic analysis

**Fig 2:**
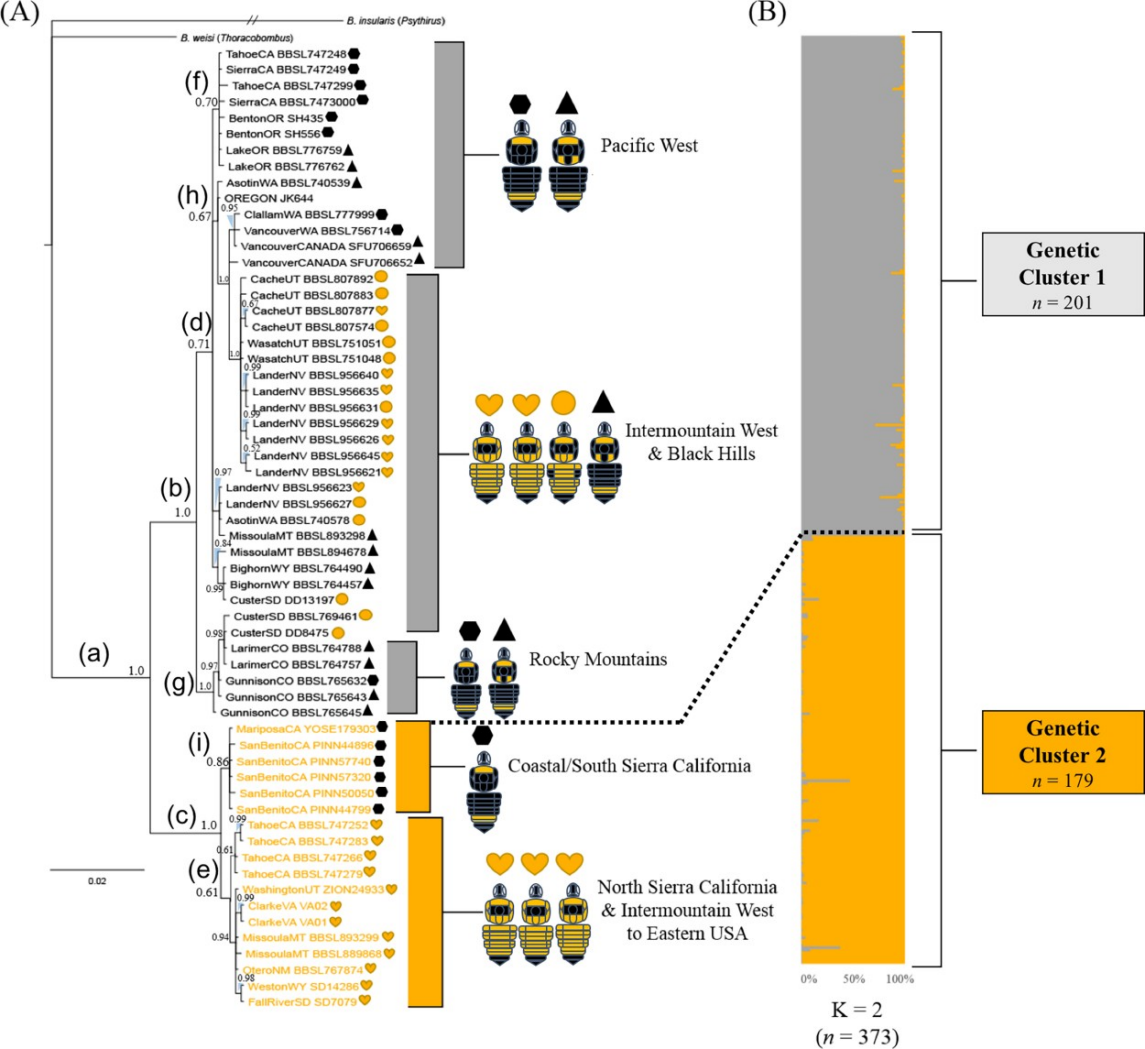
Phylogeny and microsatellite genotype assignment of *B*. *fervidus* and *B*. *californicus*. (A) Bayesian phylogeny of *B*. *fervidus* SC inferred using the fragments of three mitochondrial genes: cytochrome c oxidase I + 12s rRNA+ 16s rRNA. Values preceding each node correspond to Bayesian posterior probabilities. The scale bar indicates branch lengths in expected substitutions per site. Specimen phenotype group is mapped out with a corresponding shape and color. Phenotype 1 = black hexagon, phenotype 2 = black triangle, phenotype 3 = orange circle, phenotype 4 = orange heart. Outgroups = *B*. *weisi* (*Thoracobombus*) and *B*. *insularis* (*Psythirus*), with the branch length of the latter species truncated. Bold lowercase letters refer to the clades associated with a node preceding each lineages’ geographic distribution. (B) Fractional genotype assignment (genetic cluster) based on a Bayesian analysis of 13 microsatellite loci implemented in Structure assuming K = 2. Each horizontal bar represents a single specimen’s microsatellite genotype, where each color represents a fractional assignment to one of two genetic clusters. Colors of each fractional genotype correspond to the text color of the specimens mapped on the Bayesian phylogeny (A). Dashed line associating the phylogeny to the fractional genotype assignments of the Structure plot link the pool of corresponding individuals that were sequenced and genotyped.

Our inferred phylogeny based on the concatenated gene sequences recovered two distinct monophyletic groups with strong support (Bayesian Posterior Probability, BPP = 1.0) (Fig 2A). Our data recover a paraphyletic *B*. *californicus sensu lato* and a polyphyletic *B*. *californicus sensu stricto* and *B*. *fervidus*, but support the hypothesis that there are two phylogenetically distinct species—clade **b** and clade **c**—due to fairly long branch lengths separating them. Single gene investigations revealed similar topologies to the full evidenced set but with lower support for clades **b** and **c**, specifically, BPP_COI =_ 0.89, BPP_12s =_ 0.84, and BPP_16s =_ 0.86. All three genes contributed to the inferred Bayesian phylogeny and were retained in all analyses. Examination of sequence divergence between clades **b** and **c**, revealed the COI gene to have 861 identical sites (95.7%) with an average sequence divergence of 1.67% between clades; 16s revealed 473 identical sites (97.1%) with an average sequence divergence of 1.66%; and 12s revealed 348 identical sites (94.8%) with an average sequence divergence of 5.04%. GenBank accession numbers for the three mitochondrial gene fragments of the 64 specimens are found in S2 Table.

Species descriptions of *B*. *californicus* by Smith (1859) and *B*. *fervidus* by Fabricius (1798) did not capture the phenotype (setal color) variability associated with lineages inferred in our well supported phylogeny. While setal color variability has been documented in both species, taxonomic keys and diagnoses by Thorp et al. [20], Stephen [16], Mitchell [35], Koch et al. [17], and others do not account for the shared setal color polymorphisms uncovered in this study. Coloration patterns from the holotypes of both species have been recovered in the two clades, which impedes us from assigning taxonomic names to them. Clade **c** includes the least color variability, which has traditionally been assigned to *B*. *fervidus*. This clade contains individuals from phenotype 1 from the Coastal/South Sierra California, forming the subclade **i** (Table 1, sites 39 and 40). Within the Intermountain West + Pacific Northwest **h** clade, individuals that exhibited no signs of admixed black setae on the dorsal regions of terga two and three of the metasoma were detected, which is typically attributed to *B*. *californicus consanguineus* (Fig 2A) (Table 1, site 13) [20].

Within the respective **b** and **c** clades, we found a degree of support for geographic structuring across lineages (Fig 2A). Specifically, within clade **b,** we found strong support (BPP = 1.0) for a Rocky Mountain clade **g** as sister to the populations distributed in the Intermountain West + Black Hills and the Pacific West clade **d**. An exception was a South Dakota specimen (CusterSD, DD13197) that was found within the Intermountain West clade **d**, but it was preceded by a node with poor support (BPP = 0.71). Within clade **c**, we found strong support for the Coastal/South Sierra California clade **i** as sister to a lineage that comprises specimens distributed from North Sierra California + Intermountain West to Eastern USA clade **e**. Within clade **e** we found low support (BPP = 0.61) for the sister relationship between the North Sierra California populations and the populations that comprises the Intermountain West to Eastern USA.

### Population genetic analysis

**Fig 3:**
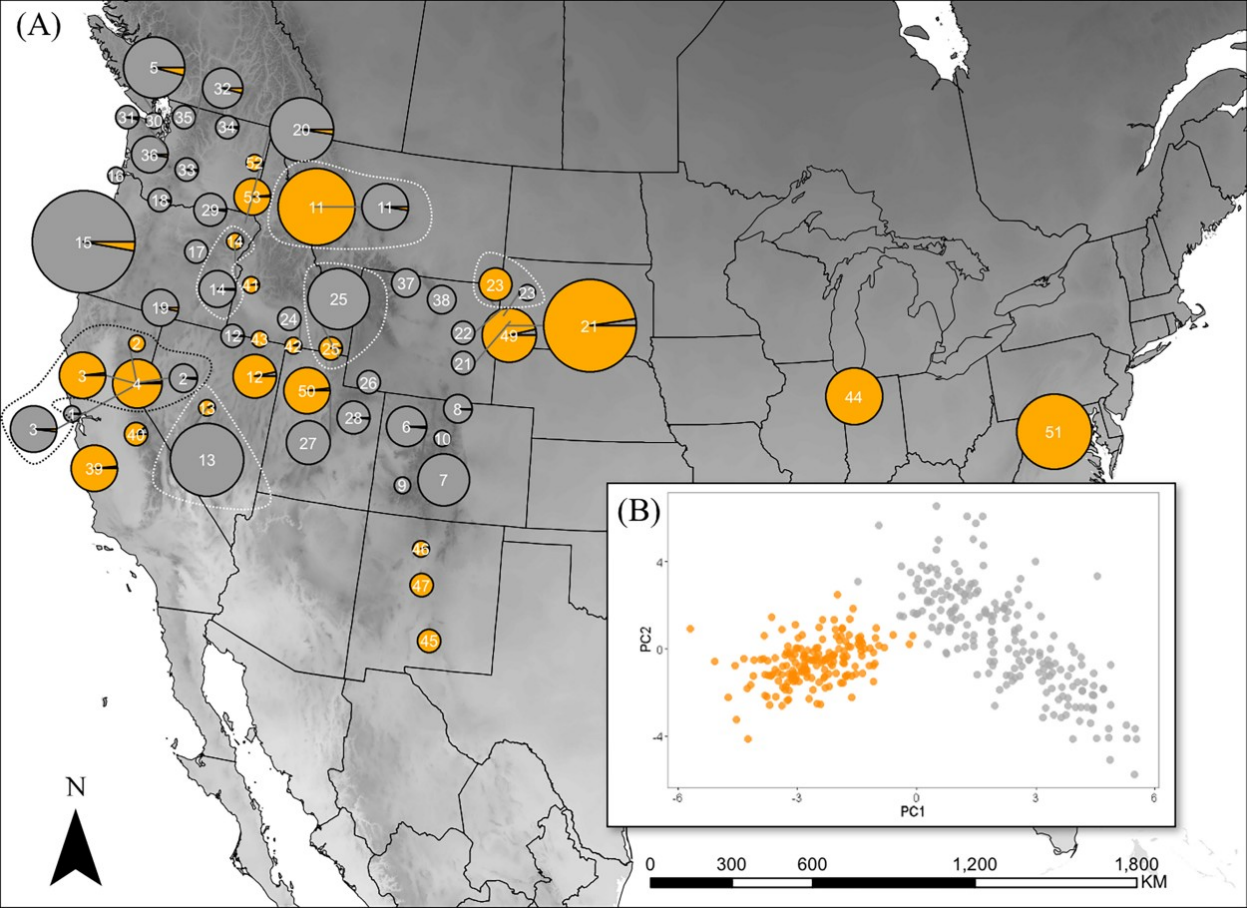
Map and principal components analysis of *B*. *fervidus* species complex microsatellite genotypes. (A) Spatial distribution of K = 2 genetic clusters, genetic cluster 1 (gray circles) and genetic cluster 2 (orange circles) inferred from a Bayesian analysis of 13 microsatellite loci implemented in Structure. The size of each circle represents the number of specimens genotyped per locality. Fractional genotypes are averaged across specimens within each genetic cluster (see Fig 2B for individual genotype assignment to a lineage). Populations enclosed by a black or white dotted polygon represent localities where genetic cluster 2 and genetic cluster 1 are geographically sympatric (*i*.*e*., Site 2: 0.92 km SSW of Sierra Valley, Sierra County, California; Site 3: 1.52 km SSW Sierraville, Sierra County, California; Site 4: 2.33 km WNW Sierraville, Sierra County, California; Site 14: 32.5 km NE Baker City, Baker County, Oregon; Site; Site 11: MPG Ranch, Bitterroot Valley, Missoula County, Montana; Site 13: Toiyabe Range, Birch Creek, site 5, Lande County, Nevada); Site 25: Logan Canyon, Cache County, Utah; Site 23: Mirror Lakes, Pennington County, South Dakota). The number at the center of each pie chart represents the field sites described in Table 1. (B) Principal component analysis of 13 microsatellite loci shared between genetic cluster 1 (gray points) and 2 (orange points).

Microsatellite genotypes corroborate the existence of two monophyletic groups inferred from the multi-gene phylogeny within the *B*. *fervidus* SC (Fig 2B). Structure analysis of the available genotypes revealed two major genetic clusters within the *B*. *fervidus* + *B*. *californicus* clade **a** (Fig 2A). The estimate of the optimal cluster is based on a Structure Harvester analysis that found the highest log likelihood of the inferred models of *K* to occur at *K* = 2 (Table 2; Mean *LnP(K|2)* = -14577.2). Significantly less explanatory power was gained by additional clusters (*ΔK* = 954.68) [55] (Table 2). Finally, at six localities in our study, we found sympatric populations of clades **b** and **c** as evidenced by distinct microsatellite genotypes (Fig 3A), and the inferred phylogeny (Fig 2A).

**Table 2:** Table of four probabilities of model fit implemented with the Evanno method associated with different values of *K* (i.e., genetic clusters) based on 13 microsatellites implemented in Structure Harvester. Bold text represents the indices that suggests the value of *K* that best predicts the microsatellite genotypes assigned in the Structure analysis.

*K*	Reps	Mean *LnP(K)*	*Ln'(K)*	*|Ln''(K)|*	*Δ K*
1	10	-15870.1	-	-	-
**2**	**10**	**-14577.2**	**1292.85**	**865.67**	**954.6799**
3	10	-14150	427.18	208.34	80.19935
4	10	-13931.2	218.84	31.18	0.906039
5	10	-13743.5	187.66	38.67	0.474636
6	10	-13594.5	148.99	99.93	1.567087
7	10	-13545.5	49.06	8.32	0.101161
8	10	-13488.1	57.38	99.03	0.945423
9	10	-13529.7	-41.65	146.24	0.348091
10	10	-13425.2	104.59	-	-

In total, 93.8% of the 373 genotyped individuals were matched to the species identifications performed by the authors (*B*. *fervidus* or *B*. *californicus*) based on the classic setal color phenotypes found in taxonomic keys and field guides (Fig 1) [16,17,20]. Of the 209 specimens exhibiting the *B*. *californicus* phenotypes (phenotypes 1, 2, and 3) (Fig 1), 5.26% were assigned to genetic cluster **2** (Fig 2B). While the genotypes of 10 specimens were assigned to genetic cluster **2**, they exhibited phenotype 1 (*B*. *californicus sensu stricto*). Eight of the specimens were collected in Pinnacles National Park and two of the specimens were collected in Yosemite National Park (S1 Table). An additional individual assigned to genetic cluster **2** exhibited phenotype 3 (*B*. *californicus sensu lato*), and was collected in Owyhee County, Idaho. Of the 172 specimens exhibiting the *B*. *fervidus* phenotype (phenotype 4), 7.3% were assigned to genetic cluster **1** (Fig 2B). While the genotypes of 12 specimens were assigned to genetic cluster **1**, they exhibited phenotype 4 (*B*. *fervidus sensu stricto*). Eight specimens were collected in the Toiyabe Range in Lander County, Nevada, one specimen was collected in the Bitterroot Valley in Missoula County, Montana, two specimens were collected in Logan Canyon in Cache County, Utah, and one specimen was collected in Guardsman Pass in Wasatch County, Utah (S1 Table).

Principal components analysis estimated 202 principal components for the 13 genetic loci used in our study. Principal component 1 explained 4% of the variance in the genotype data and principal component 2 explaining 6% of the variance in the genotype data (Fig 3B). While the number of principal components is large, visual inspection of principal components 1 plotted against principal components 2 revealed two distinct clusters associated with the genotype assignments inferred from the Structure analyses (Fig 2B). Furthermore, AMOVA results found that 14.66% of the genetic variation was partitioned among the two major genetic clusters, 14.10% among individuals within populations, and 71.24% among individuals within sites (Table 3). Overall *F*_*ST*_ among populations is 0.15 (*P* < 0.001) and *F*_*IS*_ is 0.17 (*P* < 0.001). Microsatellite genotype data is available at https://doi.org/10.6084/m9.figshare.6972518.v1.

**Table 3:** Results of Analysis of Molecular Variance (AMOVA) for genetic clusters 1 and 2 in the *Bombus fervidus* species complex (n = 330) based on allele frequencies of 13 loci.

Source of Variation	*df*	Sum of Squares	Variance Components	% Variation
Among populations	1	201.56	0.55	14.66
Among individuals within populations	356	1340.11	0.54	14.10
Within Individuals	358	965.50	2.70	71.24
Total	715	2507.173	3.79	100

*F*_*IS*_ = 0.17, *F*_*ST*_ = 0.15, *F*_*IT*_ = 0.29, (all *p* < 0.001)

To determine HWE and LD associated across populations within each genetic cluster (*i*.*e*., clade), we first separated out individuals based on genetic cluster assignment supported by Structure analysis. After partitioning the specimens by genetic clusters, we used Micro-checker to determine if any loci by population combinations exhibited evidence of null alleles or stuttering. From our analyses of population within the genetic cluster 1 (clade **b**), we elected to remove BTMS0044 as it was found to be in LD with BTERN02. Finally, BL15 and B124 did not amplify in several specimens in genetic cluster 2, and were not used in any further analyses. After the removal of problematic loci, we retained the following eight loci for further analyses with specimens assigned to genetic cluster 1: BT10, B96, BTERN02, B124, BL15, BT28, BTMS0086, BTMS0066, and the following eight loci for specimens assigned to genetic cluster 2: B126, BT10, B96, BTERN02, BTERN01, BTMS0044, BT28, BTMS0066.

**Fig 4:**
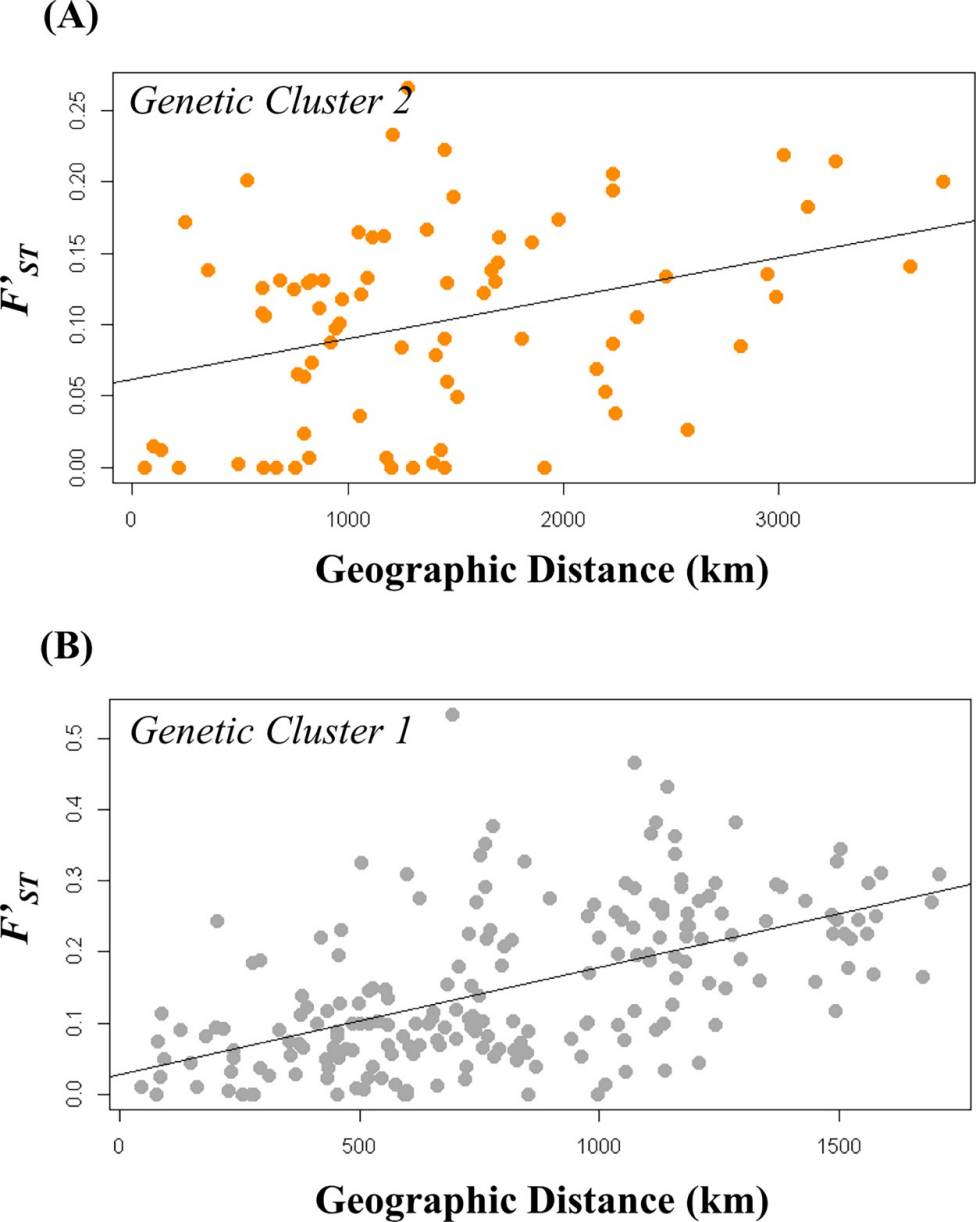
Isolation by distances (IBD) of genetic clusters 2 and 1 in the *B*. *fervidus* species complex. (A) Isolation by Distance Plot: Linearized F_ST_ between pairs of genetic cluster 2 populations compared to geographic distance. (B) Isolation by Distance Plot: Linearized F_ST_ between pairs of genetic cluster 1 populations compared to geographic distance.

Across genetic cluster 2 (clade **c**) we detected a strong effect of geographic distance on patterns of allelic fixation (Mantel Tests, *r* = 0.39, *P* = 0.03), with estimates of pair-wise linearized *F*_*ST*_ ranging from 0 to 0.26 (Fig 4A). We also detected a strong effect of geographic distance on patterns of allelic fixation within genetic cluster 1 (clade **b**) (Mantel Tests, *r* = 0.56, *P* = 0.01), with estimates of pairwise linearized *F*_*ST*_ ranging from 0 to 0.53 (Fig 4B).

## Discussion

Globally, there are more than 260 species of described *Bombus* [26]. Bumble bees are typically regarded as well studied relative to other Hymenoptera given that they represent the only extant genus in the tribe Bombini (Apidae), particularly in North America. In our study, we uncovered two well-supported lineages made up of populations that exhibit shared setal color polymorphisms across clades **b** and **c** in the *B*. *fervidus* SC (Fig 2A). Cluster assignment of 13 microsatellite loci corroborates the results of the inferred phylogeny, specifically, that two distinct genetic lineages are present in areas where the species are broadly sympatric (Figs 2B and 3).

### Phylogeny and population genetic structure

The recent and rapid diversification within the *B*. *fervidus* SC was likely driven by climate change and glacial oscillations associated with the late Pleistocene [18,61,62]. Simple pairwise examination of the average levels of divergence across COI between clade **b** and **c** is 1.67%. The observed level of divergence is below the 2% level that is often considered reflective of what delimits a species [15]. This suggests that divergence from a common ancestor likely occurred less than ~1 million years ago based on estimates of mitochondrial divergence with respect to time [63]. However, in addition to COI, we considered that distinct 16s and 12s haplotypes are characteristic of individuals associated with both clades (Fig 2). Because there are no morphological differences other than setal coloration [16,17,20], and color patterns found in the holotypes of *B*. *californicus* and *B*. *fervidus* are represented in the two clades recovered, there is no evidence to assign species names to either clade. Future studies including molecular and/or morphological data from molecular specimens and the holotypes will be crucial to establish the species boundaries within this SC.

Despite shared setal color polymorphisms in the *B*. *fervidus* SC, we reject the hypothesis that the complex is composed of a single species. However, due to the lack of evidence other than setal coloration, we cannot reject the hypothesis that *B*. *fervidus* and *B*. *californicus* are conspecific, because the type specimen of both could be included in a single lineage. Microsatellites are powerful molecular tools that have the capacity to uncover introgression between cryptic species [39,40,64]. Our microsatellite data found sympatric populations within the *B*. *fervidus* SC to be reproductively isolated, with no evidence of introgression (Figs 2B and 3). Therefore, we reject the hypothesis that *B*. *californicus* and *B*. *fervidus* produce the hybrid *B*. *californicus consanguineus* (phenotype 3).

Williams et al. [15] and Stephen [16] state that current taxonomic tools are not useful for differentiating some closely related species, including *B*. *californicus* and *B*. *fervidus*. We agree with both Williams et al. [15] and Stephen [16] that *B*. *californicus* and *B*. *fervidus* cannot be identified to species using setal color in certain parts of the geographic distribution based on the results generated in this study (Figs 2 and 3). For example, based on the data in this study, we found that specimens, which would be identified as *B*. *californicus* in southern California (sites 39 and 40) (Fig 1), could be assigned to clade **c** which are made up of populations exhibiting the “*B*. *fervidus*” phenotype (Figs 1, 2A and 3A). Furthermore, populations distributed in the Toiyabe Range in Nevada, and the Bear River Mountain Range in Utah identified as *B*. *fervidus* based on the absence of black setae on the dorsum of the metasoma [20] could be assigned to clade **b** which are made up of populations exhibiting the “*B*. *californicus*” phenotype (Fig 2A).

Given the results of our study, setal color patterns appear to be of limited taxonomic use. Despite the degree of crypsis associated within the *B*. *fervidus* SC, we assigned 89% of the *B*. *californicus* specimens used in our study to clade **b** (genetic cluster 1) with microsatellite genotypes (Fig 2A). With *B*. *fervidus*, we assigned 93% of the specimens to clade **c** (genetic cluster 2) with microsatellite genotypes based on recognized phenotypes of the SC (Figs 2 and 3). If the type specimens were samples, and the clades could be assigned to correct names, species assignment to either *B*. *californicus* or *B*. *fervidus* based on current taxonomic tools could be possible in some areas of North America. Future research on the *B*. *fervidus* SC should evaluate taxonomic characters like wing venation as it has been useful method for distinguishing between cryptic bumble bee species [65].

### Cryptic speciation and mimicry

Cryptic speciation is found in a diversity of bumble bee clades, as well as other invertebrate and vertebrate taxa. For example, species in the *B*. *lucorum* SC (*B*. *lucorum*, *B*. *magnus*, and *B*. *cryptarum*) are indistinguishable from each other using taxonomic methods of identification (*i*.*e*., setal color patterns), and can only be diagnosed to species using molecular techniques such as barcoding [22]. Müllerian mimicry is a well-documented phenomenon where sympatric species share a common aposematic phenotype to warn predators of their noxious chemical composition [66]. Like the *B*. *lucorum* SC, the *B*. *trifasciatus* SC in east Asia is another example where species identification based on setal color pattern fails to differentiate between species. Genetic divergence among the *B*. *trifasciatus* SC is hypothesized to be generated by Himalaya orogeny with Müllerian mimicry the likely factor shaping cryptic speciation among unrelated bumble bees [66]. Other cryptic species complexes among the bumble bees include the *B*. *patagiatus* and *B*. *hypocrita* SCs of Asia [67], and the *B*. *ephippiatus* SC of Mesoamerica [18,65]. Examples of cryptic speciation facilitated by Müllerian mimicry is observed in butterflies (*Heliconius* spp.) [68], spider wasps (Pompilidae) [69], velvet ants (Mutillidae) [70], and frogs (*Dendrobates* spp.) [71].

Our phylogenetic and population genetic analytical framework discovered two distinct lineages exist in the *B*. *fervidus* SC, and that they can occur in the same habitat space. The results of our study suggest that when both species are sympatric they appear to be phenotypically divergent (Figs 2, 3A), which would indicate they are not mimicking each other. However, while they can be some sympatric, some authors have suggested that *B*. *fervidus* and *B*. *californicus* inhabit different habitat niches [36]. Future research could examine how climate, mimicry, and floral niche might contribute to their ability to coexist in some portions of their range, but not in others [27,28,65,72]. For example, Pleistocene climate variation has been hypothesized to not only drive genetic divergence in *B*. *huntii*, but also differences in bioclimatic niche, and potentially in setal color variation [72]. In addition to *B*. *huntii*, there is evidence that historic climate variation has shaped patterns of genetic divergence and habitat partitioning across closely related bumble bee species, and is hypothesized to have also cascaded down to changes in setal color patterns [6,18]. In the *B*. *fervidus* SC, shared setal coloration patterns between the two clades is potentially a result of Müllerian mimicry where the model is not in the SC. For example, at MPG Ranch in Missoula, Montana (site 11), populations belonging to genetic cluster 1 (clade **b**) and 2 (clade **c**) are detected, and exhibit divergent phenotypes (Figs 1 and 3A). Other sites where *B*. *fervidus* SC species are sympatric (both genetic clusters 1 and 2 are detected), exhibit divergent phenotypes, and show no evidence for introgression include the North Sierra Nevada Mountains (sites 2, 3, 4), the Bear River Range (site 25), the Toiyabe Range (site 13), southeastern Oregon (site 14), and the Black Hills (site 23) (Fig 3A).

Among bumble bees, Müllerian mimicry is a common phenomenon, and has been documented across a diversity of communities around the globe [18,19,65,66]. For example, bumble bees in eastern North America share similar yellow and black setal coloration patterns that can make it difficult to correctly identify some individuals to species [15]. Outside of bumble bees, participation in a Müllerian mimicry ring with species of a completely different, or closely related taxonomic group is also common. For example, there is strong phylogenetic evidence that spider wasps (Pompilidae) and velvet ants (Mutillidae) have exhibit similar phenotypes when they sympatric [69].

### Conservation implications and future work

Bumble bees are well regarded for their value in agricultural ecosystems as they are efficient pollinators of a diversity of crops [4,5,73]. However, there is global concern for bumble bee decline due to economic activities associated with human growth and expansion, namely the shuffling of Hymenopteran disease due to movement of bee colonies to meet pollination demands, as well as increased urbanization and agricultural intensification [10,74–76]. *B*. *fervidus* in particular has been associated with decline at regional scales [30,32], and has been found to be highly susceptible to a suite of pathogens [77]. Despite its co-distribution with *B*. *fervidus* throughout western North America, *B*. *californicus* does not appear to be associated with high levels of pathogen incidence [74,77].

Cryptic speciation in bumble bees is well documented [22,65,67]. Application of a phylogenetic and population genetic analytical framework has revealed that using setal color patterns as a way to diagnosis species might not be useful in discriminating between closely related species [22,67]. The inability to discriminate between species due to cryptic speciation has significant implications to both biodiversity conservation and agriculture. For example, Carolan [22] discovered that *B*. *hypocrita* and *B*. *patagiatus* exhibit a similar phenotype, but are phylogenetically distinct. The widespread Russian *B*. *patagiatus* are reared by commercial greenhouse growers for pollination of food crops. Because they can be indistinguishable from *B*. *hypocrita*, which are found in Japan, there is potential for misidentification and ultimately, the unintended movement of *B*. *patagiatus* and *B*. *hypocrita* between continental Asia and Japan. The movement of non-native species or populations has the potential to displace native bumble bee species or populations [78,79], cause a reproductive disturbance with native species [80], and potentially facilitate the spread of disease [76,81,82].

A prevailing hypothesis associated with bumble bee decline includes the introduction of novel pathogens or pathogen strains [10,11]. Given the differences in pathogen prevalence between *B*. *fervidus* and *B*. *californicus*, we suggest that researchers treat the two species in the *B*. *fervidus* SC differently in the context of conservation, ecology, and evolution. Our results show that the two lineages are phylogenetically distinct (Fig 2A), with no evidence for introgression when sympatric (Figs 2B and 3). Given the pronounced genetic differences in the species, treating them as separate will allow for a more robust assessment of their conservation needs and disease profiles.

Despite the inability to identify the individuals to species based on current taxonomy, there is potential for alternative, non-destructive ways to ensure correct species identification [83,84]. Specifically, we found that microsatellite genotypes have the capacity to differentiate species, even when they are sympatric (Figs 2 and 3). While we propose that a synoptic collections of the bumble bee community be created when conducting ecological research, we have found that taking a tarsal clipping from the mid-leg for DNA extraction and subsequent genotyping is possible, which avoids sacrificing the whole individual, allowing it to continue with its contribution to the nest economy [83]. Knowledge about the evolutionary processes associated with the formation of a species is required in conservation biology [2,14,85]. In this study, we demonstrate that populations that compose *B*. *fervidus* SC lineages are cryptic, yet form well supported clades. To reduce the complex to a single species based on the inability to identify them to species using morphological traits will likely obscure the host-pathogen dynamics associated with the species, and ultimately hinder effective action on their conservation and management.

## Supporting information

S1 Table Database of specimens in the *B*. *fervidus* species complex summarizing genetic cluster assignment (*K*), phenotype/taxonomic assignment, and locality data. Specimen Voucher = unique specimen identification number; Sequence ID = unique specimen identification number associated with the GenBank accession number in S2 Table; Population ID = unique population identification number associated with each population, see Figs 1 and 3 for geographic position; Population = alternative unique population code, GenusName = genus of taxa; Phenotype (Biotype) = phenotype assignment of specimen, see Fig 1; Taxonomic Species = species assignment based on phenotype and taxonomic keys; Genotype Confirm = species assignment based on genotype (“*californicus*” = genetic cluster 1, “*fervidus*” = genetic cluster 2); Genetic Cluster = microsatellite genetic cluster assignment; Identified correctly = Yes/No statement that evaluates whether microsatellite genetic cluster assignment match taxonomic species assignment; Location Description = location description; Decimal Latitude = decimal latitude (WGS1984); Decimal Longitude = decimal longitude (WGS1984); Country = country; State/Province = state/province; County = county; K1 Assignment = genetic cluster assignment to K1 based on Structure analysis; K2 Assignment = genetic cluster assignment to K2 based on Structure analysis. Microsatellite genotype data is available at https://doi.org/10.6084/m9.figshare.6972518.v1.
(XLSX)

S2 Table GenBank accession numbers for *B*. *fervidus* SC specimens. ID # = unique identification number, Barcode = unique barcode identification number associated with S1 Table; Species = species assignment; Sub-genus = subgenus assignment, *Th*. = *Thoracobombus*, *Psy*. = *Psythirus*; Phenotype Group = phenotype assignment based on taxonomic keys; Locality = Location collected; State/Province = state/province; Decimal Latitude = decimal degrees latitude (WGS1984); Decimal Longitude = decimal degrees longitude (WGS1984); 12s = 12s Genbank accession number; 16s = 16s Genbank accession number; COI = COI Genbank accession number.
(XLSX)
